# Comparative genomics of *Aeromonas veronii*: Identification of a pathotype impacting aquaculture globally

**DOI:** 10.1371/journal.pone.0221018

**Published:** 2019-08-29

**Authors:** Hasan C. Tekedar, Salih Kumru, Jochen Blom, Andy D. Perkins, Matt J. Griffin, Hossam Abdelhamed, Attila Karsi, Mark L. Lawrence

**Affiliations:** 1 College of Veterinary Medicine, Mississippi State University, Mississippi State, Mississippi, United States of America; 2 Bioinformatics & Systems Biology, Justus-Liebig-University Giessen, Giessen, Hesse, Germany; 3 Department of Computer Science and Engineering, Mississippi State University, Mississippi State, Mississippi, United States of America; 4 Thad Cochran National Warmwater Aquaculture Center, Stoneville, Mississippi State, United States of America; Universidad Nacional de la Plata, ARGENTINA

## Abstract

*Aeromonas veronii* is a gram-negative species abundant in aquatic environments that causes disease in humans as well as terrestrial and aquatic animals. In the current study, 41 publicly available *A*. *veronii* genomes were compared to investigate distribution of putative virulence genes, global dissemination of pathotypes, and potential mechanisms of virulence. The complete genome of *A*. *veronii* strain ML09-123 from an outbreak of motile aeromonas septicemia in farm-raised catfish in the southeastern United States was included. Dissemination of *A*. *veronii* strain types was discovered in dispersed geographical locations. Isolate ML09-123 is highly similar to Chinese isolate TH0426, suggesting the two strains have a common origin and may represent a pathotype impacting aquaculture in both countries. Virulence of strain ML09-123 in catfish in a dose-dependent manner was confirmed experimentally. Subsystem category disposition showed the majority of genomes exhibit similar distribution of genomic elements. The type I secretion system (T1SS), type II secretion system (T2SS), type 4 pilus (T4P), and flagellum core elements are conserved in all *A*. *veronii* genomes, whereas the type III secretion system (T3SS), type V secretion system (T5SS), type VI secretion system (T6SS), and tight adherence (TAD) system demonstrate variable dispersal. Distribution of mobile elements is dependent on host and geographic origin, suggesting this species has undergone considerable genetic exchange. The data presented here lends insight into the genomic variation of *A*. *veronii* and identifies a pathotype impacting aquaculture globally.

## Introduction

*Aeromonas veronii* is a Gram-negative, rod-shaped, mesophilic, motile bacterium in the *Aeromonadaceae* family. It is widespread in aquatic environments across the globe [1]. Mesophilic *Aeromonas* species are a component of the normal gut or fecal flora of several hosts, such as poultry, cattle, sheep, fish, and leeches [2] [3]. Along with *A*. *hydrophila* and *A*. *caviae*, *A*. *veronii* has been associated with zoonotic motile aeromonad septicemia (MAS) in humans, terrestrial animals [1], and a number of economically important fish, including oscar cichlid [4], tilapia [5], sea bass [6], channel catfish [7], and rainbow trout [8]. Clinical signs in fish often include, but are not limited to, skin ulcers and systemic hemorrhagic septicemia [9, 10]. It is also a digestive tract symbiont in zebrafish and leeches [11–13].

A clonal pathotype of *A*. *hydrophila* is associated with significant economic losses in the Chinese cyprinid fish industry [14]. An emergent pathotype of *A*. *hydrophila* has also been attributed to catastrophic losses in the catfish industry in the southeastern United States since 2009 [15]. Phylogenomic studies have linked the etiological agents in these outbreaks, suggesting the U.S. *A*. *hydrophila* pathotype has a Chinese origin [16].

Similarly, *A*. *veronii* causes disease in fish worldwide. *A*. *veronii* has been reported from disease outbreaks in cultured channel catfish in China [7] and tilapia in Saudi Arabia [5]. It is hypothesized that *A*. *veronii* might contribute to ulcerative syndrome in Chinese longsnout catfish (*Leiocassis longirostris*) [17]. Interestingly, pairs of *Aeromonas* strains from different species are sometimes more virulent than either strain alone [18]. In agreement with this finding, *A*. *veronii* is often reported as a co-infection with other *Aeromonas* species. An *A*. *veronii*-*A*. *sobria* complex was identified from soft shell disease in turtles [19], while an *A*. *veronii*-A. *jandai* complex was reported from Nile tilapia [20].

Here we report a comparison of 41 publicly available *A*. *veronii* genomes, revealing considerable genetic exchange within the species and geographic dissemination of strain types. We also report the genome sequence of *A*. *veronii* strain ML09-123 isolated from diseased channel catfish in a commercial aquaculture pond in the southeast U.S., and we identified that it shares high identity with an isolate from Chinese aquaculture. Comparative virulence of *A*. *hydrophila* ML09-119 [21] and *A*. *veronii* ML09-123 in catfish confirmed that this clonal *A*. *veronii* pathotype has potential to cause disease in aquaculture. This work provides novel insights into the pathogenicity and epidemic potential of *A*. *veronii* in fish as well as genome variability within the species.

## Materials and methods

### Bacterial strains and data source for comparative genome analysis

*Aeromonas veronii* ML09-123 and *A*. *hydrophila* ML09-119 isolates were recovered from disease outbreaks from commercial catfish farms in Alabama and maintained in the archival collection of the Mississippi State University College of Veterinary Medicine. All *A*. *veronii* genomes (Table 1) were recovered from the NCBI genomes database (as of 2/21/2018). The GenBank accession number for the annotated *A*. *veronii* strain ML09-123 genome is PPUW00000000. Sequenced *A*. *veronii* strains used for genomic analysis originated from different hosts including human, cattle, fish, water, and sediment and different geographical locations, including U.S., China, Germany, Sri Lanka, Japan, India, South Africa, Turkey, and Greece (Table 1).

**Table 1:** *Averonii* genomes used in comparative genomic analyses.

	Strain names	Country	Source	Level	Size (Mb)	GC%	N50(bp)	Scaffolds	Plasmid	Accession	Reference
1	VBF557	INDIA	Human	Contig	4.697	58.4	19666	526		LXJN00000000.1	N/A
2	CIP 107763	USA	N/A	Contig	4.431	58.8	188049	64		NZ_CDDU00000000.1	N/A
3	pamvotica	GREECE	Surface sediment	Contig	4.919	58.1	739151	21		NZ_MRUI00000000.1	N/A
4	TTU2014-140ASC	USA	Dairy Cattle	Contig	4.676	58.6	148012	81		NZ_LKKC00000000.1	[86]
5	TTU2014-131ASC	USA	Dairy Cattle	Contig	4.675	58.6	187444	70		NZ_LKJY00000000.1	[86]
6	TTU2014-113AME	USA	Dairy Cattle	Scaffold	4.663	58.6	82585	122		NZ_LKJQ00000000.1	[86]
7	TTU2014-143AME	USA	Dairy Cattle	Contig	4.681	58.6	204478	59		NZ_LKKG00000000.1	[86]
8	TTU2014-125ASC	USA	Dairy Cattle	Contig	4.680	58.6	168256	58		NZ_LKJV00000000.1	[86]
9	TTU2014-130AME	USA	Dairy Cattle	Contig	4.679	58.6	189668	64		NZ_LKJW00000000.1	[86]
10	TTU2014-141ASC	USA	Dairy Cattle	Contig	4.680	58.6	241272	45		NZ_LKKE00000000.1	[86]
11	TTU2014-134ASC	USA	Dairy Cattle	Contig	4.680	58.6	193661	59		NZ_LKKB00000000.1	[86]
12	TTU2014-143ASC	USA	Dairy Cattle	Contig	4.678	58.6	202296	54		NZ_LKKH00000000.1	[86]
13	TTU2014-141AME	USA	Dairy Cattle	Scaffold	4.680	58.6	223907	48		NZ_LKKD00000000.1	[86]
14	TTU2014-134AME	USA	Dairy Cattle	Contig	4.681	58.6	204478	50		NZ_LKKA00000000.1	[86]
15	TTU2014-130ASC	USA	Dairy Cattle	Scaffold	4.680	58.6	247513	49		NZ_LKJX00000000.1	[86]
16	TTU2014-142ASC	USA	Dairy Cattle	Contig	4.681	58.6	247560	45		NZ_LKKF00000000.1	[86]
17	TTU2014-108AME	USA	Dairy Cattle	Contig	4.533	58.7	162342	62		NZ_LKJN00000000.1	[86]
18	TTU2014-115ASC	USA	Dairy Cattle	Contig	4.533	58.7	233487	52		NZ_LKJS00000000.1	[86]
19	TTU2014-115AME	USA	Dairy Cattle	Scaffold	4.532	58.7	238044	53		NZ_LKJR00000000.1	[86]
20	TTU2014-108ASC	USA	Dairy Cattle	Contig	4.533	58.7	208287	58		NZ_LKJP00000000.1	[86]
21	CECT 4486	USA	Surface water	Scaffold	4.411	58.9	147024	66		NZ_CDBU00000000.1	[87]
22	CCM 7244	GERMANY	Surface water	Contig	4.422	58.9	185495	74		NZ_MRZQ00000000.1	N/A
23	CB51	CHINA	Grass carp	Complete	4.584	58.6	4584103	1		CP015448	N/A
24	Hm21	FRANCE	Leech	Contig	4.685	58.7	179631	50		NZ_ATFB00000000.1	[88]
25	X11	CHINA	Wuchang Bream	Complete	4.28329	58.8	4283286	1		NZ_CP024930	N/A
26	A29	S. AFRICA	Surface water	Scaffold	4.482	58.8	165894	54		NJGB00000000.1	N/A
27	X12	CHINA	Wuchang Bream	Complete	4.77319	58.3	4773186	1		NZ_CP024933	N/A
28	AER39	USA	Human	Scaffold	4.421	58.8	1516045	4		NZ_AGWT00000000.1	*
29	LMG 13067	USA	N/A	Scaffold	4.736	58.4	147470	72		NZ_CDBQ00000000.1	N/A
30	AVNIH2	USA	Human	Contig	4.523	58.9	211774	50		NZ_LRBO00000000.1	N/A
31	AVNIH1	USA	Human	Complete	4.955	58.5	4756751	2	1	NZ_CP014774.1 NZ_CP014775.1	N/A
32	AMC35	USA	Human	Scaffold	4.566	58.5	4172420	2		NZ_AGWW00000000.1	*
33	CECT 4257	USA	Human	Scaffold	4.516	58.9	182171	52		NZ_CDDK00000000.1	[87]
34	CCM 4359	USA	Human	Contig	4.511	58.9	245067	56		NZ_MRZR00000000.1	N/A
35	B565	CHINA	Pond sediment	Complete	4.552	58.7	4551783	1		NC_015424	[89]
36	AER397	USA	Human	Scaffold	4.497	58.8	3260625	5		NZ_AGWV00000000.1	*
37	RU31B	N/A	N/A	Scaffold	4.534	58.7	73776	93		NZ_FTMU00000000.1	N/A
38	Ae52	SRI LANKA	Goldfish	Contig	4.565	58.7	158595	80		BDGY00000000.1	[90]
39	ARB3	JAPAN	Pond water	Contig	4.543	58.8	205115	63		NZ_JRBE00000000.1	[91]
40	ML09-123	USA	Catfish	Contig	4.754	58.4	299782	32		PPUW01000001	This study
41	TH0426	CHINA	Yellowhead catfish	Complete	4.923	58.3	4923009	1		NZ_CP012504.1	[92]

*Human Microbiome U54 initiative, Broad Institute (broadinstitute.org)

N/A: Not available

### Sequencing, assembly, and annotation

*A*. *veronii* strain ML09-123, recovered from channel catfish (*Ictalurus punctatus*), was sequenced using barcoded Illumina libraries prepared using a Nextera DNA Sample Prep Kit (Epicentre, Madison, WI). Paired sequences were obtained from an Illumina GAIIx sequencer using 150 bp read length (Illumina, Inc., San Diego, CA) (4,911,312 reads resulting in 118X coverage). Reads were screened using the “trim sequences” option of CLC Workbench version 6.5.1. (CLC Bio). Adaptors were removed, low quality sequences were removed, and contig creation and *de novo* assembly were conducted using CLC Workbench and Sequencher version 5.4 (Gene Codes Corporation). For annotation, the draft genome was submitted to RAST [22] and NCBI`s Prokaryotic Genome Automatic Annotation Pipeline (PGAAP).

### Pan-Core genome

Core- and pan-genome analyses were performed using EDGAR 2.0 (Blom et al. 2016) employing a generic orthology criterion based on BLAST score ratio values (SRV). BLAST scores were normalized in relation to the best hit possible. Based on the distribution of SRVs in the dataset, cutoffs were estimated as described [23]. The number of genes in the core and in the pan-genome were calculated for up to 500 random unique combinations of every number of genomes from 2 to 41. Then the number of core genes or pan-genes was plotted as a function of the number of compared genomes. An exponential decay function (core genome) or a Heaps’ power law function (pan-genome) was used to extrapolate the development of the size of the core or pan-genome.

### Phylogenetic tree and ANI calculation

To establish taxonomic positions of evaluated *A*. *veronii* genomes, a phylogenetic tree was constructed based on the complete core genome of 41 *A*. *veronii* genomes (Table 1). Gene sets of the core genome were aligned using MUSCLE [24], resulting in an alignment of 117,137 genes in total. The alignments were concatenated, yielding a multiple alignment of 2,898,457 bp per genome, 118,836,737 bp in total. This concatenated alignment was then used to compute a Kimura distance matrix, and it was used as input for the Neighbor-Joining algorithm as implemented in PHYLP [25]. A second phylogeny was constructed based on this concatenated alignment using the approximate maximum likelihood method of FastTree [26], which automatically generates local support values calculated using the Shimodaira-Hasegawa test [27]. Finally, RaxML [28] was used to construct a maximum likelihood phylogeny. To confirm this result, the optimal model for the dataset was estimated using the ModelFinder of IQ-TREE [29] using the extended model selection option. Based on the results, the GTR model with 10 rate categories for the model of rate heterogeneity was selected. Phylogenetic trees were individually created with RaxML as described above for all 2857 gene sets of the core genome. The resulting 2857 phylogenetic trees were checked for taxa showing outlier phylogenies in a significant number of individual gene trees using the rogue taxa check of RaxML [30]. No such taxa were found. The pylogeny based on individual substitution final tree was computed based on the concatenated alignment of all genes using the described models with partitioning for every codon position. The resulting tree was bootstrapped using the rapid bootstrapping method of RAxML with 100 iterations.

### Subsystems coverage

The genomes of ML09-123 and 40 other *A*. *veronii* genomes downloaded from NCBI were submitted for annotation to the Rapid Annotations using Subsystems Technology (RAST) for subsystem categorization and comparison [22]. For annotation purposes, the following criteria were chosen for the annotation pipeline: classic RAST for annotation, RAST gene caller for ORF identification, and Figfam (version release70 with automatic fix errors and fix frameshifts options).

### Secretion systems

RAST annotated protein files were submitted to MacSyFinder [11, 31], which employs systems modeling and similarity searches to identify protein secretion systems and related appendages. Analysis was conducted using the default settings using an ordered/unordered replicon dataset with linear/circular topology. All available protein secretions systems were searched with a maximum e-value of 1.0, a maximum independent e-value of 0.001, and minimal profile coverage of 0.5.

### Putative virulence factors

Putative virulence factors encoded in *A*. *veronii* genomes were identified through searches of the Virulence Factors Database (VFDB) [32]. Full datasets were downloaded from http://www.mgc.ac.cn/VFs/download.htm, and BLAST searches were conducted with all predicted protein files using CLC Genomic Workbench (version 6.5.1). Only *E-*values <1*10^−50^ were deemed significant. Results were processed with a custom Python script to extract matches with virulence genes. Hits present in almost all (N-2 or more) or almost none (2 or fewer) strains were excluded from analysis, and a binary strain 207 virulence gene matrix was constructed with 1 indicating presence and 0 indicating absence of a match with a virulence gene within a strain. The R library pheatmap [33] was used to construct a heat map from this binary matrix using default options. Distribution of predicted *A*. *veronii* virulence factors in metabolic pathways were analyzed by Blast2GO [34].

### Insertion elements

Insertion elements were determined by submitting nucleotide files of each *A*. *veronii* genome to ISsaga [35] using the following criteria: thorough local detection, yes; use a linear replicon, no; annotate cassettes given HMM profiles, no; threshold for clustering 4000 attC evalue, 1; use different HMM banks, yes; just look for attC sites, yes; maximum value attC sites, 200; minimum value for attC size, 40; use your own covariance matrix, no. Results were organized by including complete, partial, and unknown regions and removing false predicted insertions from the final results.

### Prophages

The presence of prophages in *A*. *veronii* genomes was determined using PHASTER (PHAge Search Tool Enhanced Release) [36]. Nucleotide sequences from all genomes were concatenated to serve as an input file prior to submission to the PHASTER server. Computed results were arranged into three categories: score > 90 was considered intact phage element; a score between 70–90 was deemed questionable; and score < 70 is considered incomplete phage region. Additionally, nucleotide sequences of all the identified phage regions were concatenated and aligned in MAUVE [37] to identify conserved phage regions.

### Integron identification

Identification of integrons and their components in the genomes was performed by IntegronFinder [38].

### CRISPR (Clustered regularly interspaced short palindromic repeats) and Cas (CRISPR associated genes) elements analysis

CRISPR-Cas systems and their distribution in *A*. *veronii* genomes were determined by CRISPRfinder [39] and MacSyFinder [31]. The following criteria were used for the identification of Cas elements: maximal e-value, 1.0; independent e-value, 0.001; minimal profile coverage, 0.5. Three different categories were obtained (*mandatory*, *accessory*, *forbidden*) defined as follows: mandatory elements are identifiable and ubiquitous; accessory elements could be essential but not identifiable. If the identified element is partly homologous, it is considered a forbidden element.

### Putative antibiotic resistance-related genes identification

Because the majority of the genomes are not closed, contig files were concatenated, and concatenated nucleotide files were submitted to CARD (Comprehensive Antibiotic Resistance Database) [40] to perform BLASTN search. Results were organized based on an E-value <1*10^−10^, and duplicates were removed.

### Virulence in catfish

All fish disease challenges were conducted in compliance with protocol #17–288 approved by the Mississippi State University Institutional Animal Care and Use Committee (IACUC). The approved protocol included humane endpoints, and when morbid fish met established criteria, they were immediately euthanized by immersion in tricaine methane sulfonate (MS-222). Criteria for euthanasia were loss of balance, hanging at the water surface, or non-responsiveness to external stimuli. Some of the fish died during the study as a result of the experimentally induced systemic infection due to its rapid progression. All personnel on this experiment received IACUC-approved trained in animal care and welfare by the University Laboratory Animal Veterinarian.

The relative virulence of *A*. *hydrophila* ML09-119 [21] and *A*. *veronii* ML09-123 were tested using a channel catfish challenge model. Briefly, 12 month-old channel catfish (18.2 ± 0.53 cm, 82.3 ± 6.39 g) reared indoors for disease research at Mississippi State University were stocked into twenty-seven 40-liter flow-through tanks (10 fish/tank) and acclimated for four days. Tanks were assigned randomly to two treatment groups (*A*. *veronii* and *A*. *hydrophila*) and four doses (1 x 10^4^, 1 x 10^5^, 1 x 10^6^, 1 x 10^7^) with three replicate tanks for each dose. A negative control group was also included. Water temperature was maintained at 32°C (±2) during the experiments. Fish were fed to satiation twice daily and monitored three times daily for morbidity and mortality. On the day of challenge, fish were anesthetized by immersion in tricaine methane sulfonate (MS-222), and each pre-determined dose was administered intraperitoneally (IP) in 0.1 ml volume. Fish were monitored twice daily, and mortalities were recorded for a total of seven days. The experiment was terminated when no fish mortality was observed for three consecutive days. Mean percent mortalities for each dose and treatment group were calculated and arcsine transformed. The mean transformed percent mortality for fish challenged with four doses for *A*. *hydrophila* and *A*. *veronii* was compared against sham challenged fish using the Student's t-test (p < 0.05).

## Results

### Genome features

*A*. *veronii* strains used in this study and their genome features are listed in Table 1. G+C ratios range from 58.1–58.9%. Only one strain (AVNIH1) carries a plasmid.

### Pan/Core genome analyses

**Fig 1:**
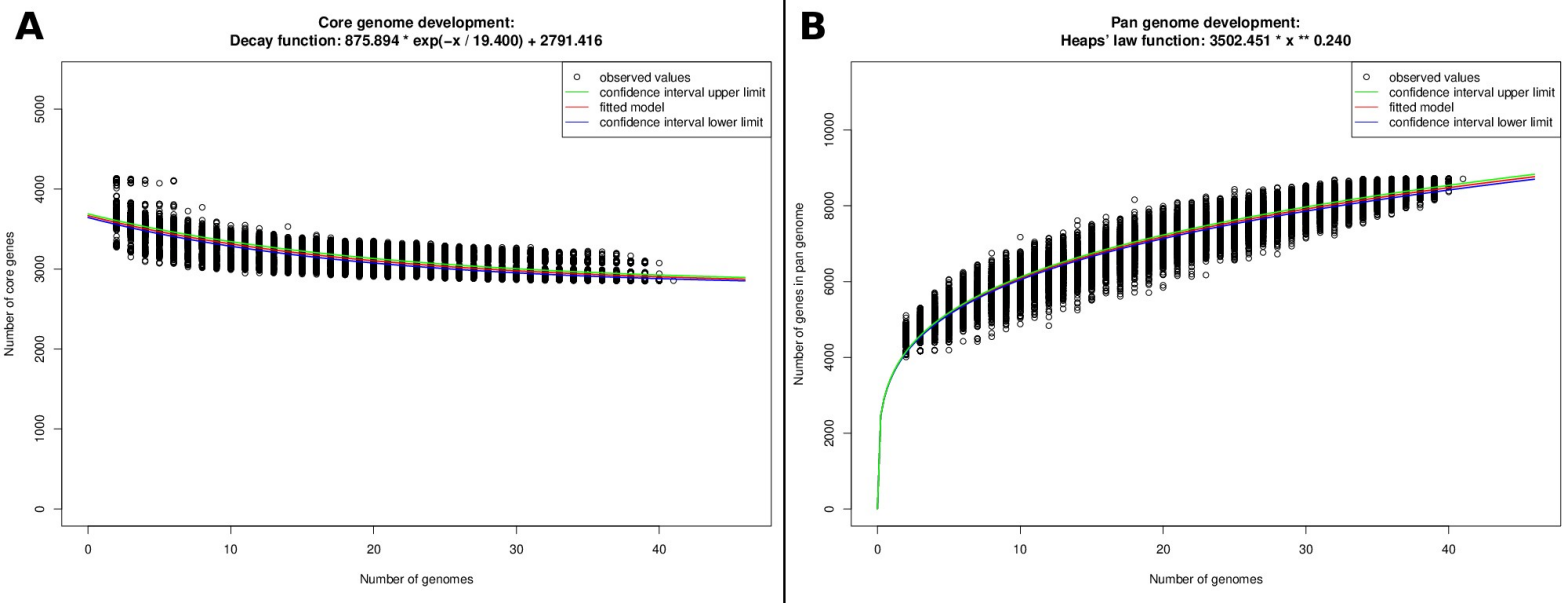
Pan vs. core genome development plot for *A*. *veronii* genomes.

Pan/core genome analysis revealed 8710 total genes in the pan-genome for 41 *A*. *veronii* genomes and 2855 genes in the core genome. The extrapolation of the core genome size (Fig 1) predicted a core genome size of 2791, which is close to the actual core genome of 2857. Extrapolation of the pan-genome showed that it is open with a growth factor γ of 0.240, which is similar to the previously reported 0.260 for *A*. *veronii* [41].

### Phylogenetic tree and ANI calculation

**Fig 2:**
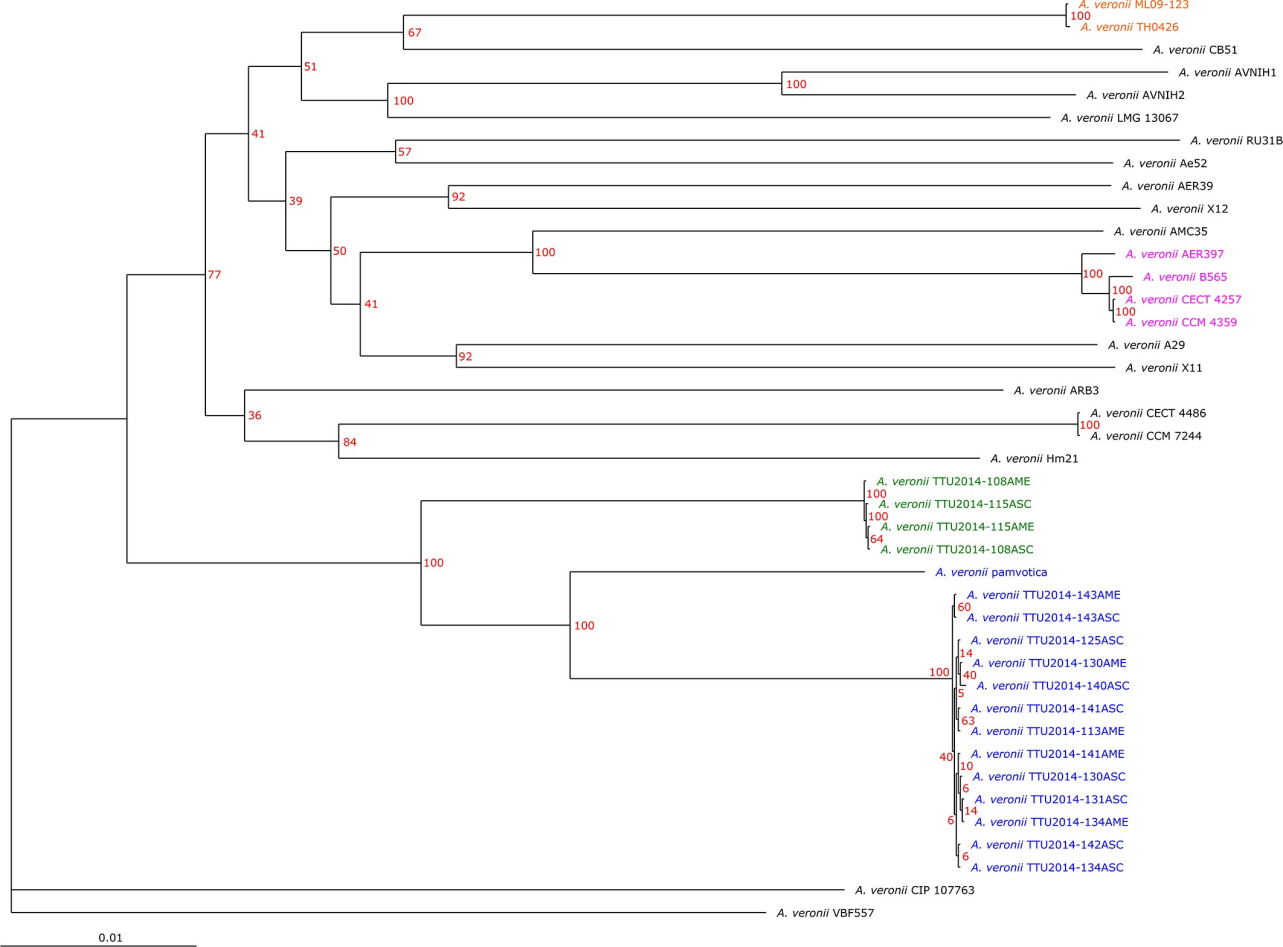
Maximum likelihood phylogeny created with RAxML. Branch labels show the branch conservation in percent of 100 rapid bootstrapping iterations. The four conserved subgroups are highlighted. The bootstrap support within the cluster of U.S. dairy cattle isolates is in part very low, most likely due to the extremely high conservation among these strains, but the separation of this cluster from the rest of the tree is very well conserved.

**Fig 3:**
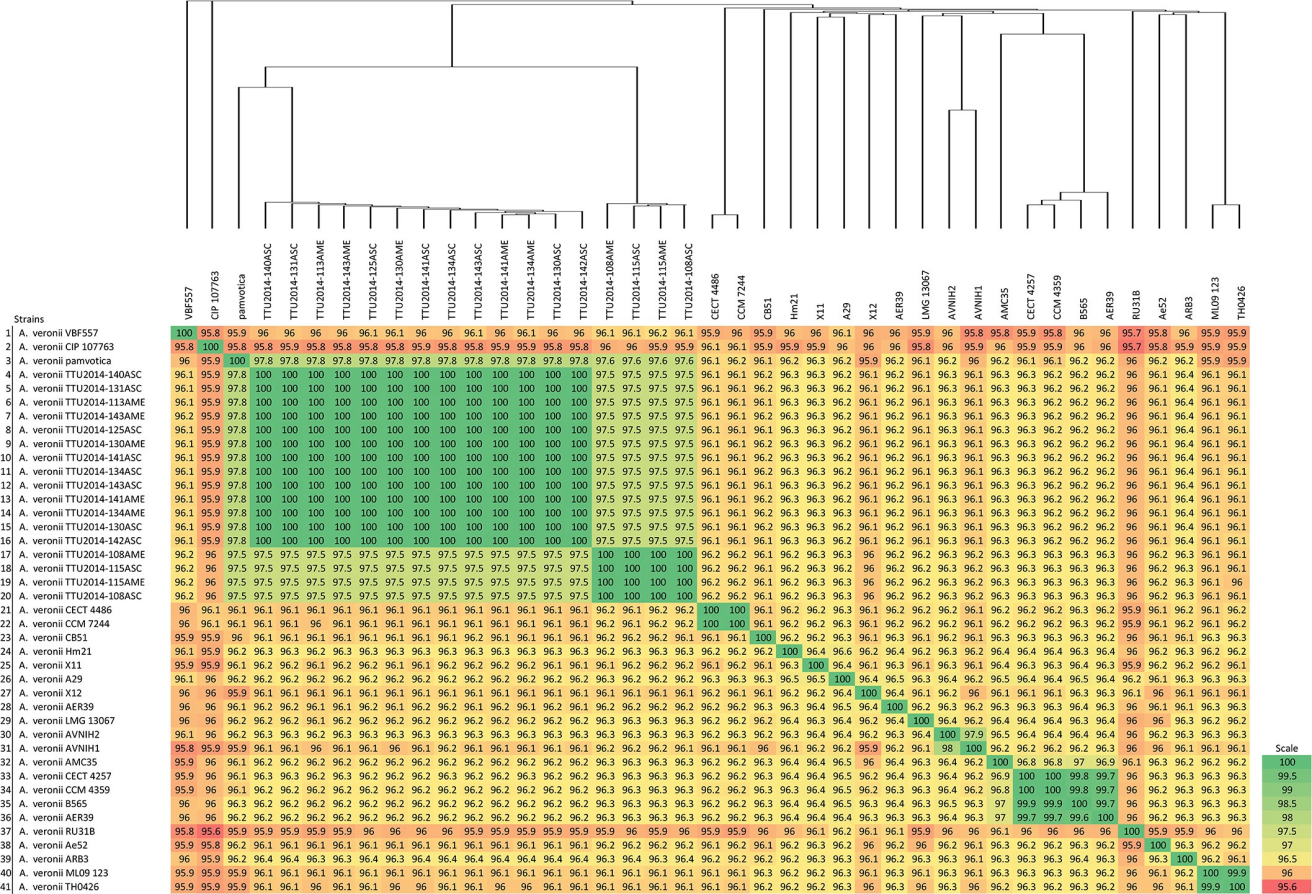
Average nucleotide identity (ANI) values of *A*. *veronii* genomes and phylogenetic tree analysis based on the core genomes. Note that branch lengths of the phylogenetic tree were reduced to fit the image.

The phylogenetic relationships of 41 *A*. *veronii* genomes were built from the complete core genome of the genomes. The phylogeny was analyzed with the distance matrix-based neighbor joining approach as well as with the maximum likelihood methods FastTree and RaxML (S1 Fig and Fig 2). Results demonstrated *A*. *veronii* to be a diverse species with many unresolved clades. Four highly conserved genetic subgroups were identified: 1) U.S. dairy cattle isolates and Greece surface sediment isolates (strain pamvotica), 2) U.S. strain ML09-123 and China strain TH0426 (catfish isolates), 3) U.S. human isolates (strains CECT 4257, CCM 4359, AER 397) and China pond sediment isolate B565, and 4) U.S. surface water and Germany surface water isolates. The remaining *A*. *veronii* isolates did not fit into these or other subgroups. These findings were confirmed and supported by Average Nucleotide Identity (ANI); ANI values in conserved branches (genetic subgroups) were above 99.91% (Fig 3).

### Subsystems coverage

**Fig 4:**
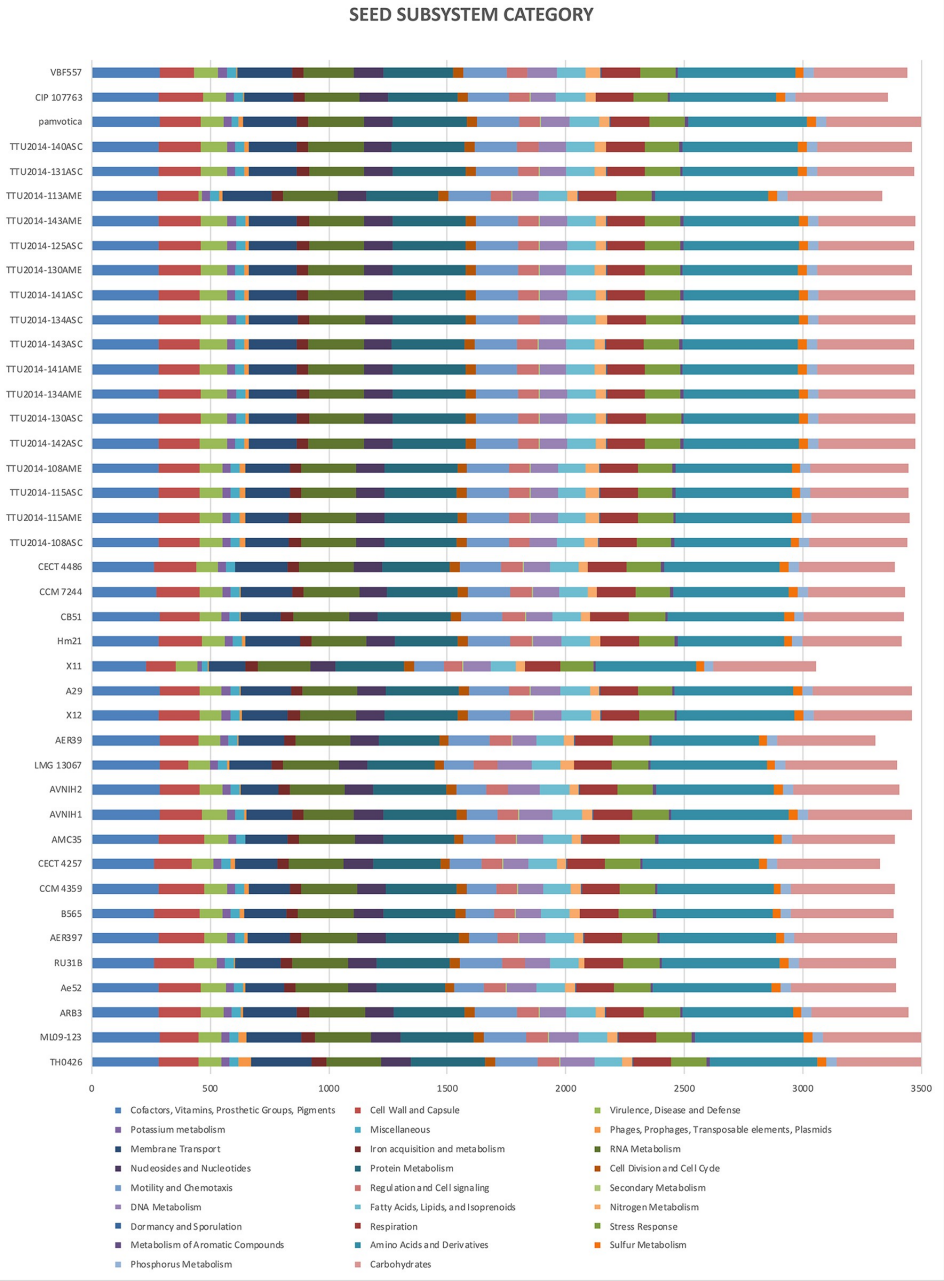
SEED subsystem category for *A*. *veronii* genomes. Comparison of functional categories in 41 *A*. *veronii* genomes based on SEED. Functional categorization is based on roles of annotated and assigned genes. Each colored bar represents the number of genes assigned to each category.

The subsystems predicted by RAST annotation are listed in Fig 4. SEED subsystem categorization analysis predicted the Chinese isolate strain TH0426 carries the most elements (3559). The most abundant system is “amino acid and derivatives” biosynthesis and utilization. The second most abundant system is “carbohydrates”, followed by “cofactors, vitamins, prosthetic groups, and pigments”. Interestingly, U.S. catfish isolate strain ML09-123 and Chinese catfish isolate strain TH0426 carry the most abundant elements in “phages, prophages, transposable elements, and plasmids”.

### Secretion systems, flagellum, TAD, and T4P

**Fig 5:**
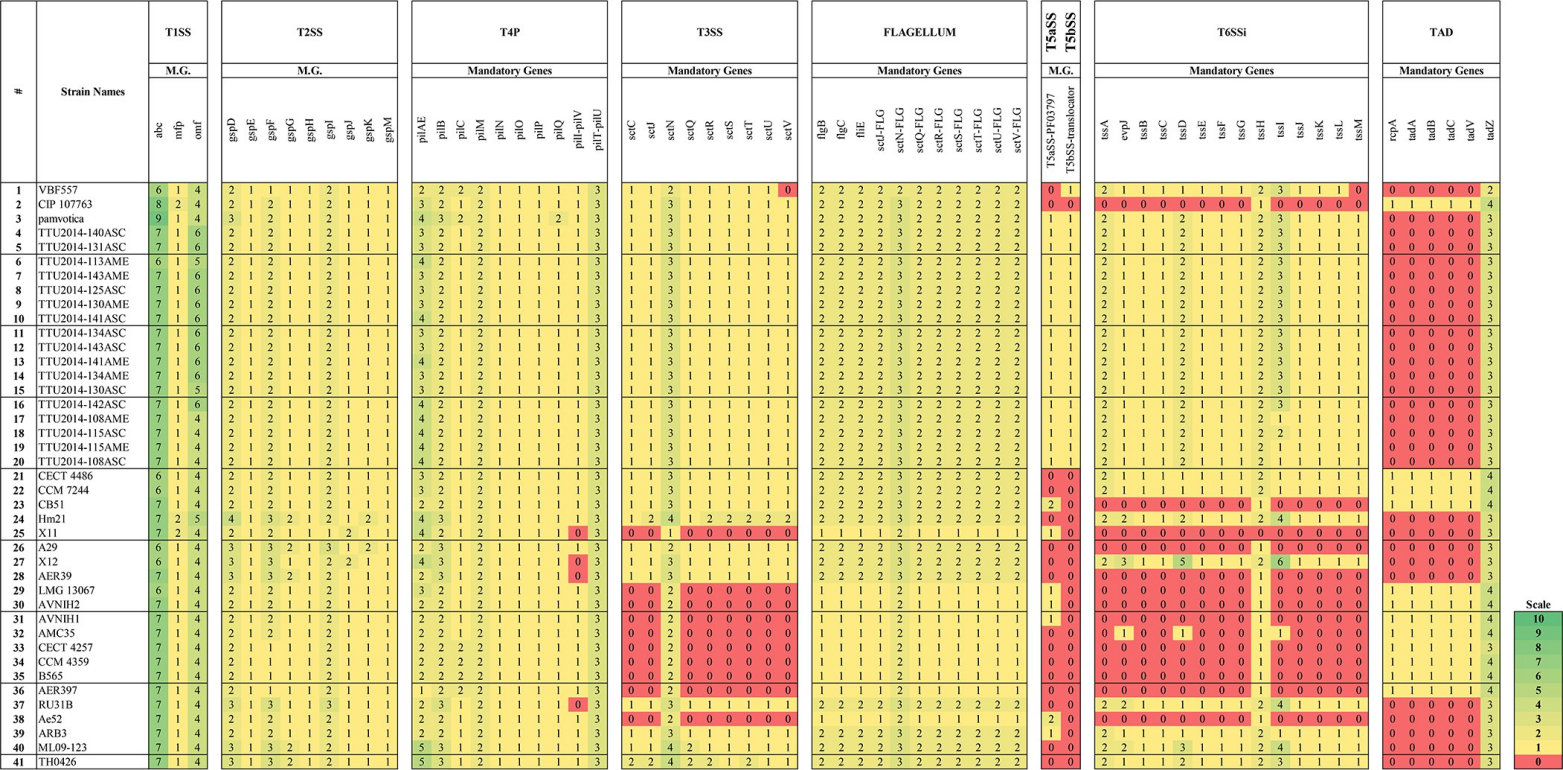
Core components of secretion systems elements, T4P, Tad, and flagella in *A*. *veronii* genomes. Numbers represent the presence of each gene. M.G.: mandatory genes.

All the evaluated genomes encode T1SS, T2SS, T4P, and flagellum core components. Some genes from these systems were absent in some strains, but this could be attributed to errors in annotation or assembly. Strains ML09-123, TH0426, RU31B, X12, and Hm21 do not encode TAD and T5SS components (Fig 5). The majority of *A*. *veronii* genomes encode T3SS. Exceptions are Sri Lanka fish isolate Ae52, pond sediment isolate B565 from China, six human isolates (, AVNIH2, AVNIH1, AMC35, CECT4257, CCM 4359, and AER397), and unknown source isolate LMG 13067 from the U.S. (Fig 5). For the T5aSS and T5bSS systems, only 17 dairy cattle isolates from the U.S. and strain pamvotica from Greece encode both systems, whereas the remaining genomes either carry one gene from each system or none (Fig 5). For the T4SS, 13 dairy cattle isolates encode eight of the T4SS-Type T accessory genes, whereas four strains (TTU2014-108AME, TTU2014-108ASC, TTU2014-115AME, and TTU2014-115ASC) do not encode this system. Strains CIP107763, AVNIH1, and pamvotica encode some of the T4SS-type T components (S1 Table). Strain AVNIH1 possesses a large plasmid that encodes several T4SS-type F elements. The genomes from strains VBF557 and pamvotica encode some of the T4SS-type F elements (S1 Table).

*A*. *veronii* genomes either carry all the mandatory genes of T6SSi or one gene, *tssH*, from this system. The presence of *tssH* in the genome of strains that do not have a T6SSi operon is not surprising because *tssH* is the most frequent T6SSi gene located outside of the T6SSi locus [11], so TssH may contribute to other functions besides T6SSi. T6SSi shows similar distribution as the T3SS with the following exceptions: strains A29, AER39, CB51 and CIP 107763 have T3SS but do not encode T6SSi system. Only 12 out of 41 genomes encode the TAD system (Fig 5).

### Virulence genes

**Fig 6:**
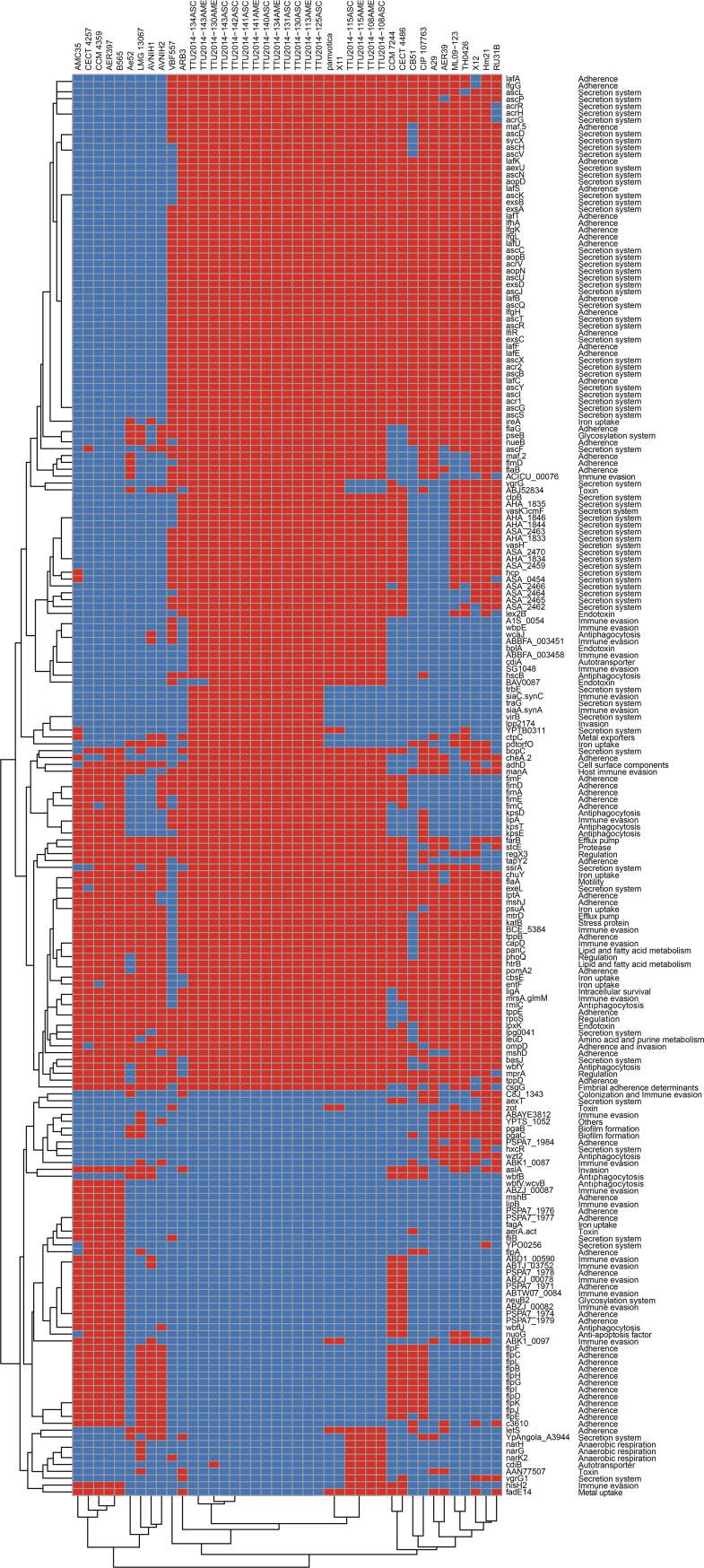
Virulence genes distribution in *A*. *veronii* genomes. Red boxes represent presence, blue boxes represent absence.

Two hundred seven putative virulence genes were identified, representing 29 categories. Secretion systems were the most common category (68 genes), followed by adherence (56 genes), immune evasion (23 genes), antiphagocytosis (11 genes), iron uptake (7 genes), and toxins (4 genes). The represented categories are shown in Fig 6. *mrsA/glmM*, *katB*, *cbsE*, *flaA*, *mtrD*, *exeL*, *rpoS*, *pomA2*, *mshJ*, *lptA*, *BCE_5384*, *ligA*, *tppB*, *lpg0041*, *lpxK*, *htrB*, *capD*, *basJ*, *tppD*, *tppE*, *panC*, *psuA*, *leuD*, *chuY*, and *ompD* genes are the most prevalent (shared by 39 *A*. *veronii* genomes), *entF*, *rmlC*, *farB*, *mprA*, *phoQ*, *and mshD* are the second most prevalent (shared by 38 *A*. *veronii* genomes), and *regX3* and *wbfY* genes are shared by 37 *A*. *veronii* genomes. The *zot* toxin gene is present in only six *A*. *veronii* genomes, including strains ML09-123 and TH0426. Some of the T6SS elements such as AHA_1837 (also known as *tssJ*) are present only in two strains (ML09-123 and TH0426).

### Insertion elements

All evaluated *A*. *veronii* genomes carry insertion elements. The number of insertion elements per genome varies from 16 to 163. Strain AVNIH1 has the highest number of insertion elements, whereas strain A29 carried the least number of insertions. Insertion families and subgroups IS1595-subtype-ISPna2, IS51, IS3, IS2, IS481, IS4, IS903, and ISL3 are encoded by a majority of the evaluated *A*. *veronii* genomes (S2 Table).

### Phage elements

Only two of the strains (CECT4486 and CCM7244) do not carry any type of phage regions, whereas the remaining genomes have at least one type of identified phage element. The general G+C content for phage elements identified within *A*. *veronii* genomes varies from 46.99–63.41%. Strains ML09-123 and TH0426 tend to have similar G+C ratios in their phage elements, ranging from 57.55–63.41%. Similarly, strain pamvotica and U.S. cattle isolates show similar G+C ratio distribution in their phage elements (S3 Table).

### CRISPR-Cas elements

The genomes of strains A29, LMG 13067, AVNIH2, AVNIH1, and AMC35 have CRISPR regions. All of the other *A*. *veronii* genomes carry questionable CRISPR regions. Cas systems are divided into three categories (Type-I, Type-II, and Type-III), with these three categories further subdivided into 10 different subcategories (Type I-A to F, Type II-A and B, Type III-A and B). Only a handful of *A*. *veronii* genomes encode these elements (TH0426, LMG 13067, AVNIH1, and AVNIH2), while the remaining genomes do not carry Cas elements (S4 Table).

### Integrons

Only two strains (AVNIH1 and Ae52) encode complete integron regions. The other *A*. *veronii* genomes either carry a cluster of *a**ttC* sites lacking integron-integrases (CALIN) or none of the integron regions (S5 Table).

### Antimicrobial resistance genes

**Fig 7:**
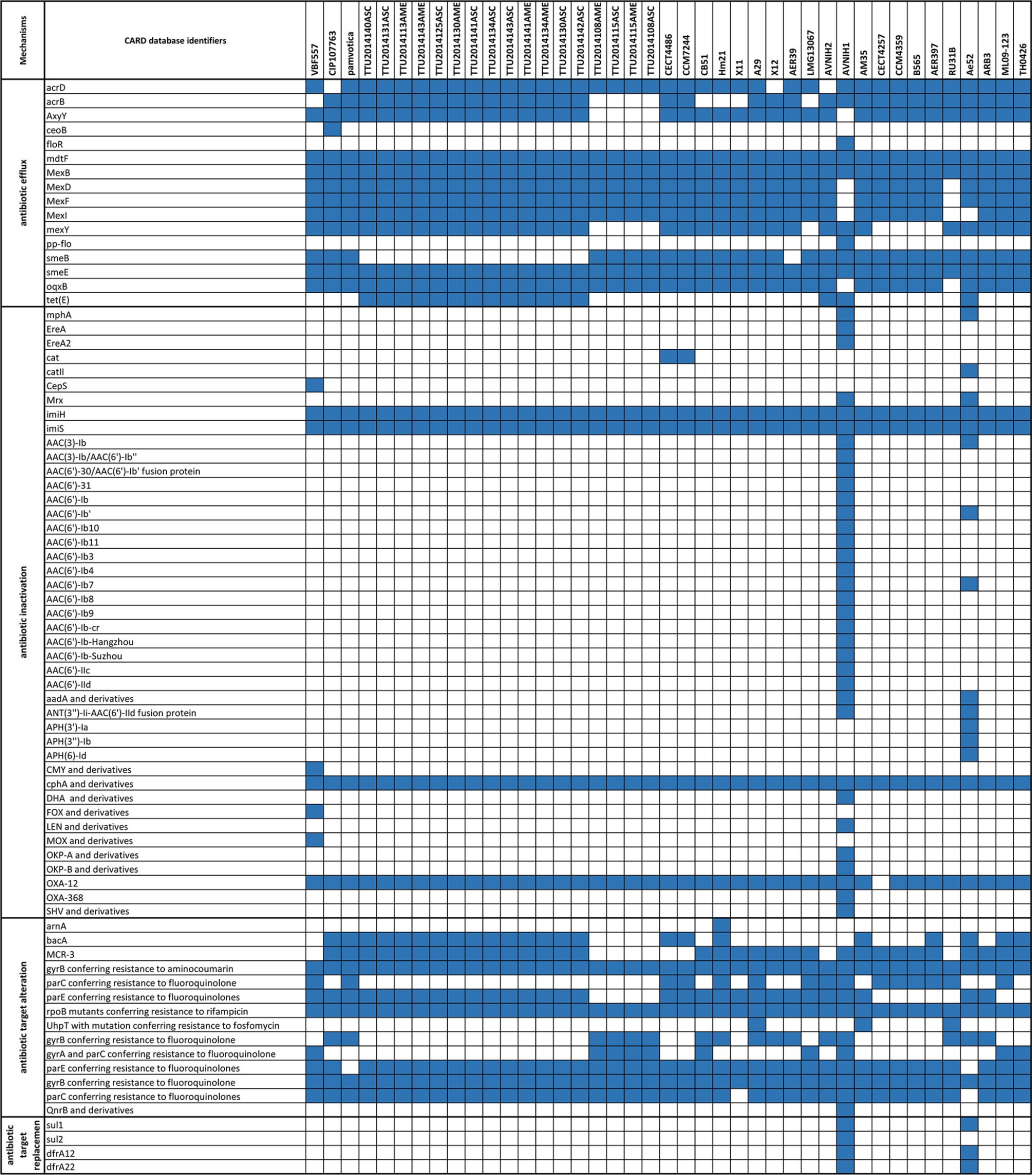
Heat map of resistome elements distribution in *A. veronii* genomes. 16s and 23s rRNA elements mutations conferring resistance to specific antibiotics are not included in Fig 7 but are provided in S6 Table.

Resistome genes and components were categorized into four categories: antibiotic efflux, antibiotic inactivation, antibiotic target alteration, and antibiotic target replacement. All of the evaluated *A*. *veronii* genomes had relatively similar resistome profiles. The AVNIH1 genome possessed the largest number of antimicrobial resistance elements. Antimicrobial resistance elements encoded by channel catfish isolate ML09-123 and Chinese channel catfish isolate TH0426 were consistent with the profiles from most of the evaluated *A*. *veronii* genomes. Antimicrobial inactivation genes *imiH*, *imiS*, and *cphA* derivates were conserved across the 41 *A*. *veronii* genomes (Fig 7 and S6 Table).

### Virulence in catfish

**Fig 8:**
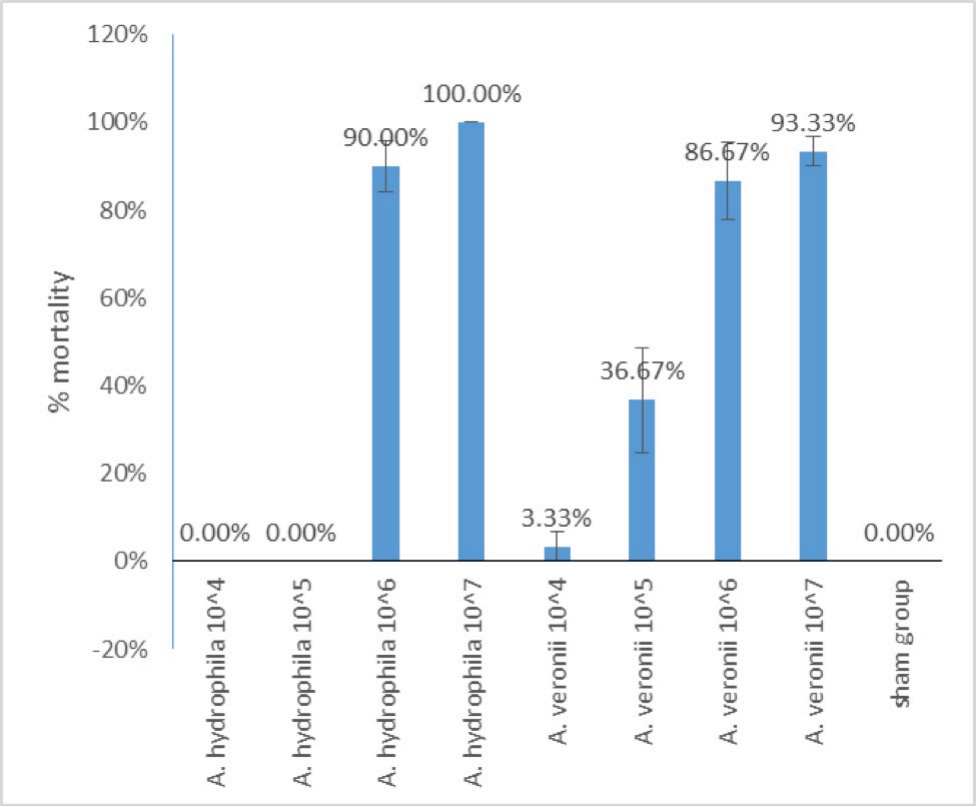
Percent mortalities in catfish challenged with *A*. *hydrophila* ML09-119 and *A*. *veronii* ML09-123.

*A*. *veronii* ML09-123 demonstrated an incremental dose response; mortalities increased with each dose: 10^4^ CFU/fish (3.33%), 10^5^ CFU/fish (36.67%), 10^6^ CFU/fish (86.67%), and 10^7^ CFU/fish (93.33%). Virulent *A*. *hydrophila* strain ML09-119 resulted in no mortalities at 10^4^ CFU/fish and 10^5^ CFU/fish, but it caused 90% and 100% mortalities at 10^6^ CFU/fish and 10^7^ CFU/fish, respectively. Results are shown in Fig 8.

## Discussion

In the current study, we sequenced the genome of *A*. *veronii* strain ML09-123 from diseased catfish in the U.S. aquaculture industry. Comparative genomics analysis suggests that ML09-123 and Chinese isolate TH0426 have a common origin. By the injection route of exposure, *A*. *veronii* strain ML09-123 is virulent in catfish and is a potential pathogen that could impact U.S. aquaculture. However, experimental infection by injection bypasses the normal mucosal barriers, so this trial does not address the potential necessity for *A*. *veronii* to require predisposing conditions to cause infection in catfish under standard aquaculture conditions.

ANI calculations can be used for an accurate digital investigation of bacterial systematics [42]. As such, ANI was used to evaluate where the *A*. *veronii* ML09-123 genome falls with respect to the publicly available *A*. *veronii* genomes. Four discrete groups were identified with a high degree of genomic homology (>99%). Interestingly, members within the groups have disparate geographic origins. Core-genome based phylogenetic analysis was consistent with and supported genetic groupings based on ANI calculations.

Pan/core genome analysis revealed a relatively low proportion of core-genome genes relative to pan-genome genes (30.9%), indicating a substantial amount of gene acquisition in *A*. *veronii* clades and strains. However, the core genome size (2272 genes) relative to the average number of protein coding genes for *A*. *veronii* (3927 genes) is 70.6%. Thus, the species has a relatively large set of core functions with highly variable sections of genome that potentially enable different environmental adaptations.

Based on SEED subsystem analysis, the Chinese catfish isolate TH0426 and U.S. catfish isolate ML09-123 carry the most abundant elements in the category “phages, prophages, transposable elements and plasmids” (Fig 4). Thus, bacteriophages appear to have contributed significantly to gene acquisition in these two strains.

Secretion systems play an important role in bacterial metabolism and pathogenesis, including host invasion, immune evasion, tissue damage, and bacterial competition [43]. Comparative secretion systems analysis revealed secretion system element distribution correlates well with the four *A*. *veronii* genetic subgroups based on ANI and core genome comparison (Fig 5). Previously, epidemic *Aeromonas hydrophila* genomes`secretion systems were evaluated [44, 45], and the presence of each secretion system varied based on the geographic location.

The T1SS is widespread in gram-negative bacteria and is involved in one-step transportation of unfolded substrates directly into extracellular space by bypassing the periplasm [46]. The T1SS is comprised of three proteins that span the cell envelope (outer membrane protein [omp], ATP-binding cassette [ABC], and the membrane fusion protein [mfp]). All evaluated *A*. *veronii* genomes possess the T1SS, but the number of mandatory elements varies depending on isolate. Because this system is often responsible for secreting tissue destructive enzymes and toxins, it may contribute to host invasion and competition. Presence of T1SS increases virulence in *Serrattia marcescens* [47] and *Vibrio cholera* [48] (Fig 5).

Similarly, all 41 *A*. *veronii* genomes encode the T2SS system, which is known for exporting hydrolytic enzymes that are important virulence factors of *A*. *hdyrophila* [49, 50]. T2SS helps *A*. *veronii* colonize the gut of leeches; in particular, export of hemolysin by T2SS is an important step for initial colonization of the leech gut [51] (Fig 5). T2SS is one of the most well-conserved secretion systems within *A*. *veronii*, suggesting *A*. *veronii* strains rely heavily on the T2SS. T2SS components are similar to Tad pilus and T4P, especially in their structural components [52, 53]. T4P can contribute to motility, adhesion, and signaling [54]. All the mandatory genes of T4P are carried by the evaluated *A*. *veronii* genomes.

Flagellum is evolutionarily similar to T3SS. We identified that all the *A*. *veronii* genomes encode the mandatory genes for flagellum. Interestingly, eleven of the *A*. *veronii* genomes do not encode T3SS (Fig 5). Seven of the nine human isolates lack T3SS, suggesting this system may not be critical for mammalian virulence, whereas Silver et al. reported the importance of T3SS in *A*. *veronii* strain HM21R virulence in mice [55]. T3SS regulatory network [56] and effector proteins [57] have been investigated in *A*. *hydrophila*. In *Aeromonas salmonicida*, T3SS is linked to immunosuppression in fish [58]. *A*. *veronii* uses T3SS to protect itself from leech immune cells [55]. SctN is a component of the T3SS; interestingly, the gene encoding this protein is present in one to four copies in all the evaluated *A*. *veronii* genomes. SctN contributes to preparing substrates for export [59] and regulates transfer of effector proteins across host cell membranes [60].

T4SS facilitates exchange of mobile genetic elements between bacteria. In *Aeromonas culicicola*, T4SS enables conjugative genetic material transfer between bacteria [61]. In our analyses, some *A*. *veronii* strains encode components of two different types of T4SS, type T and F (S1 Table). Presence of T4SS in some of the evaluated *A*. *veronii* genomes correlates with the number of insertion elements present in corresponding genomes, which suggests that T4SS might be affecting the genome structure of some *A*. *veronii* strains by promoting gene uptake.

T5SS is one of the most widespread, simplest, and abundant systems in bacterial genomes. It is divided into five subtypes (T5aSS-T5eSS) [11], and it utilizes a unique mechanism for secreting substrates, including toxins and receptor proteins [43]. It is known for secreting a large number of proteins, even more than T2SS, and some of its substrates are well-known to contribute to virulence [62, 63]. In our analysis, all the cattle isolates and strain pamvotica encode mandatory genes of T5aSS and T5bSS, but the other evaluated genomes carry only one or none of these mandatory genes (Fig 5). Therefore, even though this system is known for contributing to adherence, biofilm formation, virulence, toxin export, and mediating cell-to cell interactions in other pathogens, T5SS may not be important in *A*. *veronii* virulence.

There are three type of T6SS: T6SSi, T6SSii, and T6SSiii. The majority of the *A*. *veronii* genomes encode the T6SSi system, which is well-known for contributing to virulence and the ability to interact with environment, host, and competitor bacteria [64]. In *A*. *hydrophila*, T6SS contributes to virulence and translocates the effector protein Hcp into eukaryotic cells, which can modulate activation of host immune cells [65, 66]. Interestingly, only one T6SSi gene (*tssH*, also known as *clpV*) is conserved across all the evaluated *A*. *veronii* genomes (except strain X11). TssH is an ATPase implicated in the recycling of other T6SS components, TssB/TssC, which are part of the tubular sheath [67, 68]. It was proposed that TssH is used to provide energy to contract the tubular sheath [69]. However, because *tssH* is present in *A*. *veronii* genomes that lack genes encoding core T6SS components, TssH may have another important role. In our analysis, we could not identify a pattern for presence of this system based on genetic subgroups, geographic location, or host. However, presence of T6SS in *A*. *hydrophila* genomes varies based on the geographic location [70].

The Tad system is essential for colonization, biofilm formation, and virulence of some species [71]. For example, tight-adherence genes are required for virulence of *Actinobacillus actinomycetemcomitans* [72]. The majority of *A*. *veronii* genomes (30 out of 41) do not encode this system. Interestingly, all the genomes carry at least two, some as many as four, copies of *tadZ*, which encodes one of the core components of TAD system. While functionality of this component remains unclear, it is thought to be peripherally linked with the inner membrane [71].

*Aeromonas spp*. are important pathogens in humans and other organisms. Surface polysaccharides, iron binding systems, exotoxins and other extracellular enzymes, secretion systems, adhesins, and flagella are some of the main factors contributing to *Aeromonas* virulence [73]. Interestingly, *A*. *hydrophila* and *A*. *veronii*-*Aeromonas sobria* groups contain more virulence genes than the *Aeromonas caviae*-*Aeromonas media* group, suggesting that *A*. *hydrophila* and *A*. *veronii*-*A*. *sobria* may be more adapted to a pathogenic lifestyle [74]. *A*. *veronii* isolates from diseased Gibel carp have some variation in the virulence genes they carry, but both encode and express several secreted enzymes [75]. Our virulence database search showed that secretion systems and their components are the most prevalent putative virulence elements in the *A*. *veronii* genomes, followed by adherence and immune evasion (Fig 6).

Insertion elements are important in bacterial genome evolution, contributing by gene inactivation and structural changes to the genome [76]. Insertion elements can affect virulence by causing expansion of flanking regions, gene inactivation and decay, genome rearrangement and reduction, and incorporation of additional genes [77]. In our analysis, two genetic subgroups (U.S. and China catfish isolates and U.S. cattle and Greece surface sediment isolates) encode similar insertion families, whereas the remaining *A*. *veronii* isolates demonstrate a more scattered IS distribution pattern. T4SS contributes to DNA uptake or exchange from other bacteria. In our analysis, strains pamvotica and AVNIH1 encode either all the accessory T4SS genes or some of them, and these two strains encode the largest number of insertion elements (S2 Table). Thus, in *A*. *veronii* there appears to be a correlation between number of insertion elements and presence of T4SS.

Bacteriophages can mediate horizontal gene transfer, including virulence genes, from one bacterial strain to another. Specifically, prophage regions can provide specific mechanisms for bacterial attachment, invasion, and survival capabilities [78]. In our panel of isolates, all *A*. *veronii* genomes have phage elements except two (strains CECT4486 and CCM7244). U.S. catfish isolate ML09-123 and China catfish isolate TH0426 have different numbers of bacteriophage elements, but they share one intact phage region. We did not identify a conserved phage element present in all 41 *A*. *veronii* genomes (S3 Table).

CRISPR-Cas systems consist of two main components, CRISPR array and CRISPR associated genes (Cas), which are separated from each other by spacers [79]. They provide protection against viral predation and foreign DNA invasion (lysogenic bacteriophages, plasmids, or transposons) [80]. Our results showed that all the evaluated *A*. *veronii* genomes have either confirmed or questionable CRISPR elements, but only strains TH0426, LMG 13067, AVNIH1, X11 and AVNIH2 encode Cas elements along with CRISPR regions (Table 2).

**Table 2:** General features of CRISPR-Cas loci in 41 *A*. *veronii* genomes.

Strain names	CRISPR regions and their features					Cas elements availability	
	Confirmed	Questionable	Direct repeat length	Number of spacers	CRISPR length	Cas-TypeI	CAS-TypeIII
VBF557	-	5	24–34	1	96–107	-	-
CIP 107763	-	2	25–34	1	96–105	-	-
pamvotica	-	6	23–34	1–4	72–235	-	-
TTU2014-140ASC	-	5	24–40	1	96–118	-	-
TTU2014-131ASC	-	5	24–40	1	96–118	-	-
TTU2014-113AME	-	6	25–42	1–2	96–258	-	-
TTU2014-143AME	-	5	24–40	1	96–118	-	-
TTU2014-125ASC	-	5	24–40	1	96–118	-	-
TTU2014-130AME	-	5	24–40	1	96–118	-	-
TTU2014-141ASC	-	5	24–40	1	96–118	-	-
TTU2014-134ASC	-	5	24–40	1	96–118	-	-
TTU2014-143ASC	-	5	24–40	1	96–118	-	-
TTU2014-141AME	-	5	24–40	1	96–118	-	-
TTU2014-134AME	-	5	24–40	1	96–118	-	-
TTU2014-130ASC	-	7	24–40	1	96–118	-	-
TTU2014-142ASC	-	5	24–40	1	96–118	-	-
TTU2014-108AME	-	4	24–39	1	96–124	-	-
TTU2014-115ASC	-	4	24–39	1	96–124	-	-
TTU2014-115AME	-	4	24–48	1	96–133	-	-
TTU2014-108ASC	-	4	24–39	1	96–124	-	-
CECT 4486	-	7	23–37	1	76–115	-	-
CCM 7244	-	7	23–37	1	79–124	-	-
CB51	-	3	45–55	1	147–166	-	-
Hm21	-	3	24–35	1	99–113	-	-
X11	1	5	23–48	1–58	97–3572	cas3-TypeI	-
						cse1-TypeIE	
						cas7-TypeIE	
						cas5-TypeIE	
						cas2-TypeIE	
A29	2	4	24–43	1–9	81–684	-	csx16-TypeIIIU
							cas1-TypeII
X12	-	7	24–42	1	81–133	-	-
AER39	-	4	24–34	1	105–107	-	-
LMG 13067	1	3	32–45	1–42	106–2804	cas1-TypeIC	-
						cas4-TypeI-II	
						cas7c-TypeIC	
						cas8c-TypeIC	
						cas5c-TypeIC	
AVNIH2	1	5	23–45	1–72	80–4430	cas2-TypeIE	-
						cas1-TypeIE	-
						cas5-TypeIE	
						cas7-TypeIE	
						cse2-TypeIE	
						cse1-TypeIE	
						cas3-TypeI	
						cas6-TypeIE	
AVNIH1	1	7	23–46	1–4	80–250	cas3-TypeI	-
						cas3-TypeI	
AMC35	1	8	23–51	1–5	80–371	-	-
CECT 4257	-	6	23–53	1	95–158	-	-
CCM 4359	-	4	23–53	1	95–158	-	-
B565	-	5	23–53	1	95–158	-	-
AER397	-	7	24–53	1	81–127	-	-
RU31B	-	4	23–42	1	86–121	-	-
Ae52	-	3	24–45	1	106–117	-	-
ARB3	-	5	24–35	1	96–108	-	-
ML09-123	-	7	23–48	1	98–133	-	-
TH0426	-	7	23–42	1	99–139	cas6-TypeIF	-
						csy3-TypeIF	

Integrons are a major type of genetic element resposible for spread of antibiotic resistance genes. They consist of two main components: 1) integron-integrase (*intI*) and its promoter region (P_intI_) and 2) attachment site of the integron (*attI*) and constitutive promoter (Pc) that integrates gene cassettes at *attI*. For a functional integron, only these core elements (*intI* gene and *attI* integration site) are required [81]. Gene cassettes typically have an open reading frame (ORF) with flanking *attC* genes, which mediate integration into integrons. Complete integron elements include integrase and at least one *attC* region. Incomplete integrons include ln0 elements that consist of integrase without *attC* sites and clusters of *attC* lacking integron-integrase (CALIN) [38, 81–83]. Strain AVNIH1 has one large plasmid that carries three integrons. On the other hand, strain Ae52 encodes a complete integron in its chromosome. Many of the *A*. *veronii* genomes either carry only one, two, or none of the CALIN elements in their genome.

Strain AVNIH1 has more putative antimicrobial resistance genes than other *A*. *veronii* genomes we analyzed, which could be the result of encoding three integrons. Our antimicrobial resistance analysis showed a majority of the putative antimicrobial resistance elements are shared by all the *A*. *veronii* genomes with some exceptions (Fig 7) (S6 Table).

Virulent *A*. *hydrophila* (vAh) is an important cause of MAS outbreaks in catfish aquaculture in the southeast U.S. By the experimental injection route of exposure, *A*. *veronii* strain ML09-123 had similar virulence as vAh strain ML09-119. One important difference is that ML09-119 did not cause any mortality at doses of 10^4^ and 10^5^ CFU/fish, while ML09-123 caused a dose response that correlated with mortalities at the intermediate doses (Fig 8). This suggests the two pathogens use different mechanisms to regulate virulence gene expression. A quorum sensing regulatory mechanism for virulence genes could explain the mortality pattern for vAh. N-acylhomoserine lactone (AHL)-mediated quorum sensing mechanism is important for regulating virulence of *A*. *hydrophila* SSU [84]. Virulence of *A*. *hydrophila* and *A*. *salmonicida* towards burbot (*Lota lota* L.) larvae is also controlled by quorum sensing [85].

In conclusion, our comparative *A*. *veronii* genomes analyses shows that *A*. *veronii* strain ML09-123 is highly similar to China strain TH0426. This observation was confirmed by ANI, core genome comparison, and subsystem category distribution (particularly secretion systems, insertion elements, and putative antibiotic resistance genes). Our results confirm that *A*. *veronii* is a potential pathogen in *Ictalurus* catfish and that its virulence by the experimental injection route is similar to vAh. Thus, strains ML09-123 and TH0426 appear to be inherited from a common ancestor strain and affect aquaculture in both China and the U.S. We also found that pan genome is still open and the core genome may reduce as further strains are added to the repertoire. However, approximately 30% of *A*. *veronii* genomes show considerable variation, particularly in putative virulence genes, which contributes to a relatively large pan-genome for the species.

## Supporting information

S1 Table Secretion systems distribution in *A*. *veronii* genomes. (XLSX)

S2 Table Insertion elements distribution in *A*. *veronii* genomes. (DOCX)

S3 Table Prophages distribution in *A*. *veronii* genomes. (XLSX)

S4 Table CRISPR regions and Cas elements in *A*. *veronii* genomes. (XLSX)

S5 Table Integron elements distribution in *A*. *veronii* genomes. (XLSX)

S6 Table Antibiotic resistance elements distribution in *A*. *veronii* genomes. (XLSX)

S1 Fig Maximum likelihood phylogeny created with FastTree. (DOCX)
