# Supplementary material for: Comparative genomics of Aeromonas veronii: Identification of a pathotype impacting aquaculture globally
Source: PLoS One. 2019 Aug 29;14(8):e0221018. doi: 10.1371/journal.pone.0221018 (PMC6715197; doi:10.1371/journal.pone.0221018)
Supplement: S2 Table — (DOCX) [file pone.0221018.s002.docx]

Supplementary file-2- Insertion elements distribution in *A. veronii* genomes

Reference : Varani AM, Siguier P, Gourbeyre E, Charneau V, Chandler M. ISsaga is an ensemble of web-based methods for high throughput identification and semi-automatic annotation of insertion sequences in prokaryotic genomes. Genome Biol. 2011;12(3):R30
