# Supplementary material for: Comparative genomics of Aeromonas veronii: Identification of a pathotype impacting aquaculture globally
Source: PLoS One. 2019 Aug 29;14(8):e0221018. doi: 10.1371/journal.pone.0221018 (PMC6715197; doi:10.1371/journal.pone.0221018)
Supplement: S1 Fig — (DOCX) [file pone.0221018.s007.docx]

**Maximum likelihood phylogeny created with FastTree.** Branch labels show local support values calculated using the Shimodaira-Hasegawa test. The four conserved subgroups are highlighted with colors.
